# Alcohol Hangover Increases Conflict Load via Faster Processing of Subliminal Information

**DOI:** 10.3389/fnhum.2018.00316

**Published:** 2018-08-21

**Authors:** Nicolas Zink, Wiebke Bensmann, Christian Beste, Ann-Kathrin Stock

**Affiliations:** Cognitive Neurophysiology, Department of Child and Adolescent Psychiatry, Faculty of Medicine, Technische Universität Dresden, Dresden, Germany

**Keywords:** alcohol hangover, cognitive control, conflict, cue, flanker effect, subliminal prime

## Abstract

The detrimental effects of acute alcohol intoxication and long-term alcohol (ab)use on cognition are well-known. Yet, only little is known about the cognitive effects of an acute alcohol hangover, even though it might affect executive functions associated with workplace performance or driving skills. Given that alcohol hangover may increase the speed of information accumulation, we assessed the behavioral effects of conflict load (induced by a subliminal prime) on cognitive control, as assessed via the Flanker effect. We employed a counter-balanced within-subject design, where *n* = 25 healthy young males were tested once after a sober night and once after a night of experimentally induced heavy drinking of cheap brandy/red wine (2.6375 g alcohol per estimated liter of body water within 2–3 h). Alcohol hangover neither increased the cognitive conflicts induced by consciously processed distractors alone (i.e., the Flanker effect), nor modulated conflict adaptation (i.e., the Gratton effect). Instead, hangover potentiated the detrimental effects of conflicting subliminal primes on top-down cognitive conflicts. This effect was likely due to an increase in the speed of information accumulation from visual stimuli and the resulting increase in subliminal conflict load induced by incompatible primes. We further found the size of this effect to be positively correlated with age and subjective sleepiness during the hangover state, but the hangover effect remained significant even after correcting for those covariates. We further found no correlation of the behavioral effect with the subjective overall rating of hangover symptoms or the maximal breath alcohol concentration reached during prior intoxication. Taken together, our findings suggest that alcohol hangover may affect cognitive performance due to an increase in non-conscious processing of visual distractors. While the size of this effect might increase with age and sleepiness, it is not entirely dependent on those covariates and not necessarily related to subjective ratings of general hangover symptoms/impairment.

## Introduction

In many parts of the world, alcohol (ethanol) is a widely and regularly consumed substance. Due to the detrimental effects of excessive consumption on both physical and mental health, alcohol (ab)use causes a wide range of socioeconomic and health-related issues that cause great financial and societal costs (WHO, 2014). In this context, alcohol-induced changes of cognitive functioning take a special role, as they may not only contribute to the development and maintenance of alcohol use disorders (AUD) (Stock, 2017), but may also impair workplace productivity and safety (Bush and Lipari, 2013). It has been found that cognitive control and executive functions generally tend to be most severely affected by alcohol (e.g., Crews and Boettiger, 2009), but there is a huge difference in how well long-term and short-term consequences of excessive alcohol consumption have been researched: Cognitive changes and impairments in AUDs have received much attention because they typically cause high societal costs and some of the cognitive impairments have proven to permanently persist even after the discontinuation of chronic alcohol consumption (Stock, 2017). There is also a growing number of studies on the cognitive effects of acute alcohol intoxication, which has repeatedly been shown to increase risky and/or aggressive behavior and to substantially increase the risk of injury or accidents (e.g., Sønderlund et al., 2014). The effects of alcohol hangover on cognitive functioning have however been largely neglected, even though a hangover does not only significantly impair subjective ratings of well-being, but may also reduce diving safety (Verster et al., 2014) and has been estimated to produce huge economic and societal costs (for review, please see Stephens et al., 2008).

Alcohol hangover is defined as “the combination of mental and physical symptoms, experienced the day after a single episode of heavy drinking, starting when blood alcohol concentration approaches zero” (van Schrojenstein Lantman et al., 2017c). Even though the picture is much less clear than for AUD or acute intoxication, it has repeatedly been suggested that alcohol hangover also affects memory, executive functions and various measures of attention to a greater degree than rather automated processes (for review, please see Stephens et al., 2008). Yet still, the cognitive effects of an acute, binge-like alcohol intoxication and an alcohol hangover might qualitatively differ due to neuropharmacological effects associated with both states: Using a drift diffusion modeling approach on a moving dots paradigm, a recent study of our work group could show that the drift rate, which reflects the speed of information accumulation for response selection, is significantly slower/decreased when intoxicated, but seems to be significantly faster/increased after a night of heavy experimentally induced drinking (both compared to a sober state) (Stock et al., 2016b). While this study did not directly quantify neurotransmitter levels, it could be speculated that this differential effect might be due to the fact that the ethanol metabolite acetaldehyde, which is typically increased during a hangover, parallels the dopaminergic effects of ethanol, but has the opposite effect on GABAergic signaling (i.e., ethanol increases GABAergic signaling, acetaldehyde decreases it) (Foddai et al., 2004; Melis et al., 2007; Enrico et al., 2009; Correa et al., 2012; Martí-Prats et al., 2013; Stock et al., 2016b). Further studies will however be needed to underpin that claim in humans *in vivo*.

The faster accumulation of response-relevant information from visual input might hypothetically help to compensate some of the hangover-associated cognitive side effects. It could however also increase cognitive conflicts arising from distracting visual input, especially when target and distractors share stimulus features that are relevant for responding. The reason is that those features may trigger stimulus-response (S-R) associations that promote automatic processing and response formation (Klapp, 2015). Given that this mechanism could potentially underlie reports of increased distractibility/attentional lapses (McKinney et al., 2012b) and resulting safety issues, including driving performance (Verster et al., 2014), we set out to investigate whether the increased processing speed of potentially task-relevant stimulus information might also increase cognitive conflict load and thereby impair behavioral performance. In line with recommendations made by Stephens et al. (2008), we decided to assess (selective) attention with the help of a Flanker paradigm, where distractors and target share response-relevant stimulus features. As processing speed does however not necessarily affect the performance in this task, we also wanted to include an experimental paradigm with a distractor that is only briefly presented. We therefore chose to conduct a conflict paradigm adapted from Boy et al. (2010). It combines a masked subliminal prime with consciously perceived flankers and therefore allows to assess the effect of cognitive conflicts on behavioral performance both in the presence and in absence of consciously initiated, top-down control processes (Stock et al., 2016a). Due to the extremely short presentation time of the prime (only 30 ms), differences in the speed of information accumulation should most likely show in this aspect of task performance. Previous studies using the same paradigm had shown that incompatible subliminal primes seem to induce a conflict load that impairs subsequent behavior (reflected by a larger flanker effect following incompatible primes) (Stock et al., 2016a; Gohil et al., 2017) and an acute alcohol intoxication seems to diminish priming effects in this task (Stock et al., 2017). Against this background, we expected that that an alcohol hangover should produce larger priming effects and/or larger flanker effects following an incompatible prime, as compared to a non-hungover sober state.

Given that various factors like the type and amount of consumed beverages as well as the accuracy of recalling a previous night's alcohol consumption might be flawed in naturalistic studies, where participants are asked to come to the lab after a typical night out, we decided to take an experimental approach where hangover symptoms are experimentally induced by the administration of a standardized amount of brandy and/or red wine the night before the hangover assessment and quantified with the help of subjective ratings on a 0 to 10 Likert scale. In order to minimize the effects of inter-individual variation, we chose a balanced within-subjects design where each subject was tested once sober/non-hungover and once hungover/the night after drinking (for details of the procedure, please refer to the Methods section).

## Materials and methods

### Participants

A group of *n* = 25 healthy young male participants aged 18–27 were recruited to participate in the study. All participants had normal or corrected-to-normal vision, and had been recruited using flyers and online ads at the local University (TU Dresden, Germany).

None of the participants reported any somatic, neurological, or psychiatric diseases. Female subjects were generally excluded from the study due to concerns of the local ethics commission about the potential risk of pregnancy. All participants reported at least one episode of pronounced ethanol intoxication within the past 12 months. None of the participants had risky drinking habits as defined by alcohol use disorder identification test (AUDIT; Babor et al., 2001) scores between 1 and 15 (mean 10.1; SD 2.7), which indicates low-to-moderate risk of alcohol addiction/abuse as well as a small likelihood of pronounced homeostatic alcohol tolerance. All participants gave written informed consent before starting the experiment and were reimbursed with 80 € after their participation. The study was approved by the ethics committee of the Faculty of Medicine of the TU Dresden and conducted in accordance with the Declaration of Helsinki.

### Experimental design and induction of hangover

The study was conducted using a within-subject design where each subject was tested two times (once in a sober/non-hangover state and once hungover), with the order of appointments balanced between subjects (subjects were randomly assigned to either the “sober first” or the “hangover first” group). Between the sober and the hangover state, there was a delay of at least 48 h but no more than 7 days. All participants were asked to refrain from the use of caffeine, nicotine, guanine and all other stimulant or sedative substances as well as pain medication within the last 4 h before the start of each experimental session and were asked to not consume any alcohol the night before their sober appointment.

**Figure 1:**
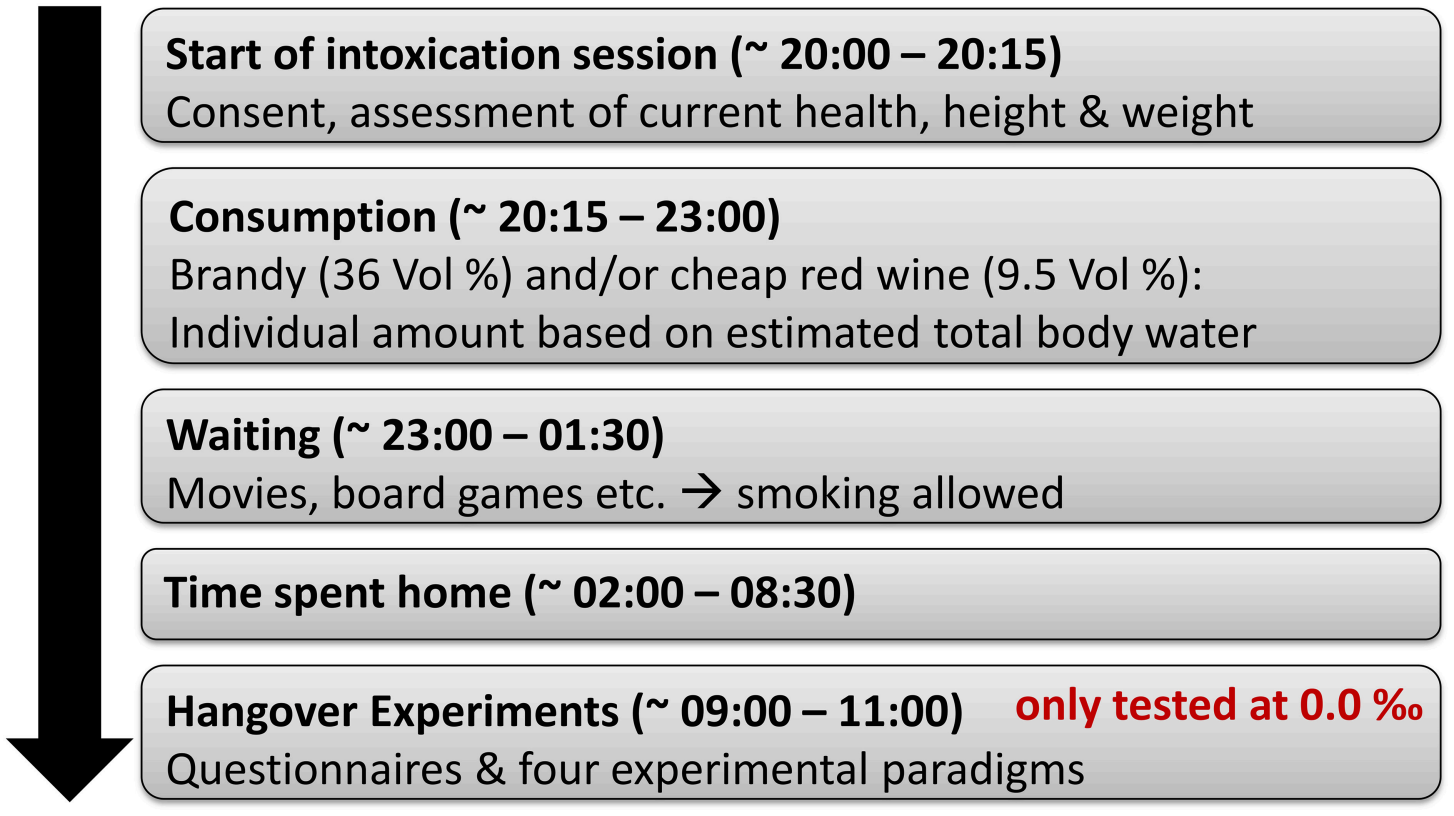
Illustration of the hangover induction procedure. In order to induce alcohol hangover symptoms, we invited our participants to the laboratory on Friday or Saturday evening at 20:00. After obtaining written consent, a quick assessment of any recent health concerns, height and weight, an individually determined amount of alcohol (2.6375 g of alcohol per estimated liter of body water) was consumed on a full stomach from ~ 20.15 to 23:00. Until 01:30, participants were kept in the laboratory and asked to provide BAC measures in half-hour intervals, starting 30 min after their last sip of alcohol. Participants were then brought home via taxi services and invited to show up in the laboratory at 09:00 the next morning for the hangover appointment. The data collection of the hangover appointment was however not started before participants had reached a BAC of 0.00‰.

As the mixture of vodka and orange juice, which is considered to be the gold standard for acute intoxication studies, likely produces only little to no hangover symptoms (Verster, 2008), the alcohol administration procedure used in this study differs from the protocol used in acute intoxication studies of our group (e.g., Stock et al., 2015, 2016b, 2017) in several aspects: We only provided alcoholic beverages with a high congener content (brandy and red wine), as those are more likely to provoke hangover symptoms (Verster, 2008) and tend to produce a more severe hangover (Rohsenow et al., 2010). We furthermore asked participants to eat before starting their consumption, as piloting had shown that those drinks could not be consumed on an empty stomach in sufficient quantities without frequent adverse side effects like nausea, stomach aches, and vomiting. In order to increase the amount of ingested alcohol and congeners without exceeding comparatively safe limits of about 1.6‰, we furthermore stretched a slightly larger amount of alcohol (2.6375 g of alcohol per estimated liter of body water instead of the 1.9781 g/l used in acute intoxication studies) over a substantially longer consumption period (3 h instead of 30 min). Lastly, we slightly restricted sleep duration the night before the hangover appointment as total sleep time seems to be inversely associated with hangover severity (van Schrojenstein Lantman et al., 2017a). For details on the hangover procedure, please see Figure 1.

We used a version of the equation by Widmark (1932) and Watson et al. (1980) to calculate an individual amount of alcohol in grams for each participant to attain a maximal possible breath/blood alcohol concentration (BAC) of 2.0‰ (mg/g) and an approximate mean peak BAC of 1.6‰ in case the entire amount was consumed at once or on an empty stomach (i.e., with a resorption deficit of ~20%). At an assumed absorption deficit of 30–40% due to the full stomach and enforced drinking delays required in this study (the access to alcohol was restricted by the experimenters so that the participants could not consume the alcohol in <2 h), a mean BAC of about 1.2‰ can be reached with a neglectable likelihood of exceeding a BAC of 1.6‰. The tool used for the calculation of individual amounts can be viewed in the Supplementary Material. In short, it provides an estimate of the total body water in liters (based on sex, age, height, and weight) and indicates how many grams of alcohol need to be diluted in the body water to reach a certain intoxication level. Based on the alcohol content of each beverage, this is then translated into the amount of beverage (in ml) that needs to be consumed.

The participants were allowed to choose whether they wanted to drink cheap brandy (36 Vol %), cheap red wine from a tetra pack (9.5 Vol %), or a mixture of both. The amount of consumed alcoholic beverages was limited by the individually determined amount of alcohol in grams (see Supplementary Material), but the participants were free chose whether they preferred their beverages pure, chilled with ice, or diluted with various caffeine-free soft drinks (ginger ale, orange lemonade, coke). Participants were not allowed to consume caffeinated beverages together with the alcohol as this might increase subjective sleep quality and alertness during the hangover appointment (Rohsenow et al., 2014). Access to those alcohol-free beverages as well as to tap water was not restricted. Furthermore, all participants had access to free snacks (tortilla chips and wine gum) and were allowed to smoke during the intoxication session as smoking while drinking has been suggested to promote hangover symptoms (Jackson et al., 2013; Epler et al., 2014). Breath alcohol levels were measured using the “Alcotest 3000” breath analyzer following the instructions of the manufacturer (Drägerwerk, Lübeck, Germany) 30, 60, 90, and 120 min after the participants had finished their last alcoholic drink.

Before the start of both the sober and hangover appointments, breath alcohol levels were assessed again. Both appointments could not start unless the participants presented with a BAC of 0.00‰. Participants, who had not (yet) reached this value, were asked to stay in the lab and BAC was assessed every 20 min. When they had reached a BAC of 0.00‰, we waited for an additional 30 min before starting the data collection as blood alcohol levels might take slightly longer than breath alcohol levels to return to zero (Verster et al., 2017).

### Questionnaires

At the start of each appointment, participants were asked to indicate how many hours they had slept in the night prior to that appointment and to rate their overall hangover severity as well as 23 hangover-specific symptoms listed by van Schrojenstein Lantman et al. (2017c) on a Likert scale ranging from 0 (no symptoms) to 10 (extreme symptoms).

During the sober appointment, participants were furthermore asked to provide sociodemographic data and fill in Beck's depression inventory (BDI; Beck et al., 1961) in order to assess potential depressive symptoms which might interfere with cognitive performance. They were furthermore asked to fill in the anxiety sensitivity index (ASI, Reiss et al., 1986), which assesses how aversive the participants deem physical symptoms of stress and anxiety. As hangover symptoms like sweating, trembling, dry mouth, stomach pain or nausea partly overlap with anxiety symptoms, we assessed this trait to obtain a rough estimate of how aversive physical hangover symptoms might be for the participants.

### Experimental paradigms

Subjects were seated at a distance of 57 cm from a 17 inch CRT monitor and were asked to respond using the two “Ctrl” buttons on a USB keyboard. During the entire experiment, participants had to rest their fingers on the response buttons. “Presentation” software (Version 17.1 by Neurobehavioural Systems, Inc.) was used to present stimuli and record the behavioral responses in both paradigms.

While our main research question was focused on the effects of subliminal primes and how the conflict load they induce modulates the flanker effect, we had to make sure that we were indeed assessing the detrimental effects of conflict induced by incompatible primes, and not merely the beneficial effects of compatible primes (please refer to discussion for details). We therefore started each appointment with a plain Eriksen Flanker paradigm that was characterized by quite large/salient arrowhead stimuli, a high proportion of compatible trials and a high time pressure in order to ensure a maximum flanker effect in the absence of any priming effects. After this, the participants were asked to spend 30 min working on a different, conceptually unrelated mental rotation task, the results of which have not yet been analyzed or published. This interruption was intended to minimize carryover effects between the two tasks. After the rotation task, the participants were subjected to our combined conflict paradigm, which assesses the effect of subliminally induced conflict load on the flanker effect.

#### Task 1: eriksen flanker task

For investigating attentional and conflict monitoring processes in the absence of any priming effects/conflict load, we used a classic Eriksen Flanker task (Kopp et al., 1996) that has already yielded stable flanker effects in previous studies of our group (Zhang et al., 2017; e.g., Mückschel et al., 2017).

Before the start of the experiment, subjects completed a supervised task practice. Each subject had to practice at both appointments until he was able to comply with the task instructions and had no further questions.

**Figure 2:**
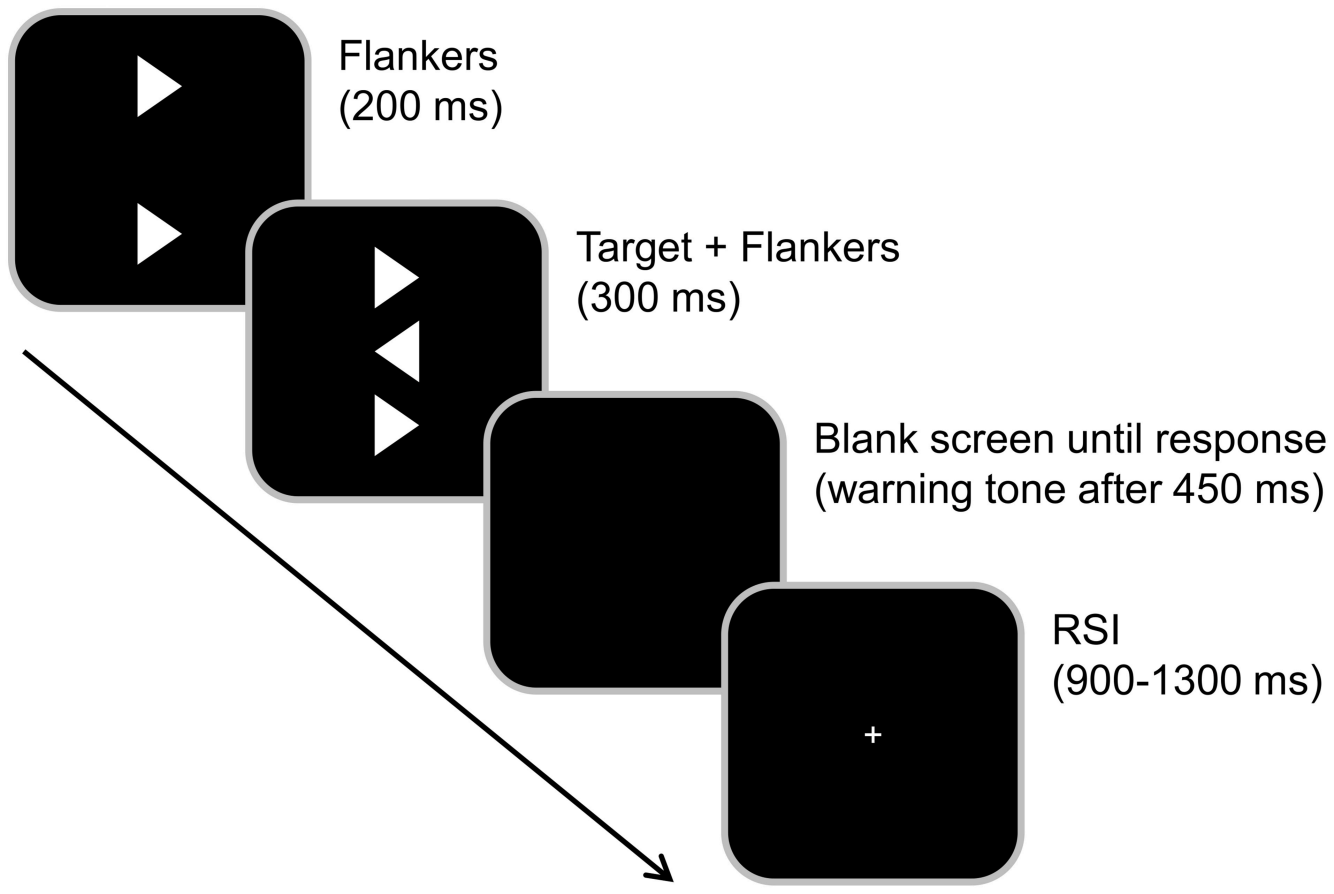
Illustration of the Eriksen Flanker task. Each trial began with the presentation of two flanker stimuli both pointing either to the left or right. After 200 ms, the target-stimulus was then presented in the center for 300 ms and simultaneously switched off together with the flankers. Flankers and target pointed either in the same (compatible) or in the opposite (incompatible) direction. The subjects had to determine the direction of the target-stimulus (the central arrowhead) by pressing the left and right Ctrl-buttons. Compatible (75%) and incompatible stimuli (25%) were presented randomly. The response-stimulus interval was randomly varied between 900 and 1,300 ms.

Every trial began with a 200 ms presentation of two vertically aligned flankers (white arrowheads) in the center of a black screen. The target (a third, identical arrowhead presented in between the flankers) was added to the visual array and presented together with the flankers for another 300 ms (see Figure 2 for illustration). After this, the screen turned black until the participant responded. The subjects had to determine the direction of the target stimulus (the central arrowhead) by pressing the left and right Ctrl buttons on a regular computer keyboard using their left and right index fingers. Compatible (75%) and incompatible stimuli (25%) were presented randomly. To exert time pressure, a warning tone was presented if the subjects did not respond within 450 ms. Additionally, participants were encouraged to respond as quickly and accurately as possible. After the response was given, a white fixation cross was presented in the center of the screen during the response-stimulus interval (RSI), which randomly varied between 900 and 1,300 ms.

#### Task 3: combined conflict paradigm

We used a task based on an experimental paradigm developed by Boy et al. (2010), which is identical to the version used in previous studies of our group (Stock et al., 2016a, 2017; Gohil et al., 2017). In short, this paradigm allows to investigate the conflicts induced by subliminally and consciously perceived distractors, as well as their interaction (i.e., the effect of subliminal conflict load on the top-down processing of consciously perceived stimulus input).

Before the start of the experiment, subjects completed a supervised task practice, where they received feedback about the accuracy of every response (no such feedback was provided during the later data collection). Each subject had to practice at both appointments until he was able to comply with the task instructions and had no further questions. On both appointments, participants were given the same instruction as during a previous study investigating acute intoxication effects using the same task (Stock et al., 2017): “*You will see three pictures in a very rapid succession. They will indeed be so fast that you might not be able to properly tell them apart, but this is fine. Your only task will be to use the right and left Ctrl buttons on the keyboard in front of you to indicate where the middle arrow you see in the last picture is pointing. Please press the left button with your left index finger when the middle arrow points left, and the right button with the right index finger if the middle arrow points right. Importantly, ignore the outer arrows as they may sometimes point in the opposite direction*.”

**Figure 3:**
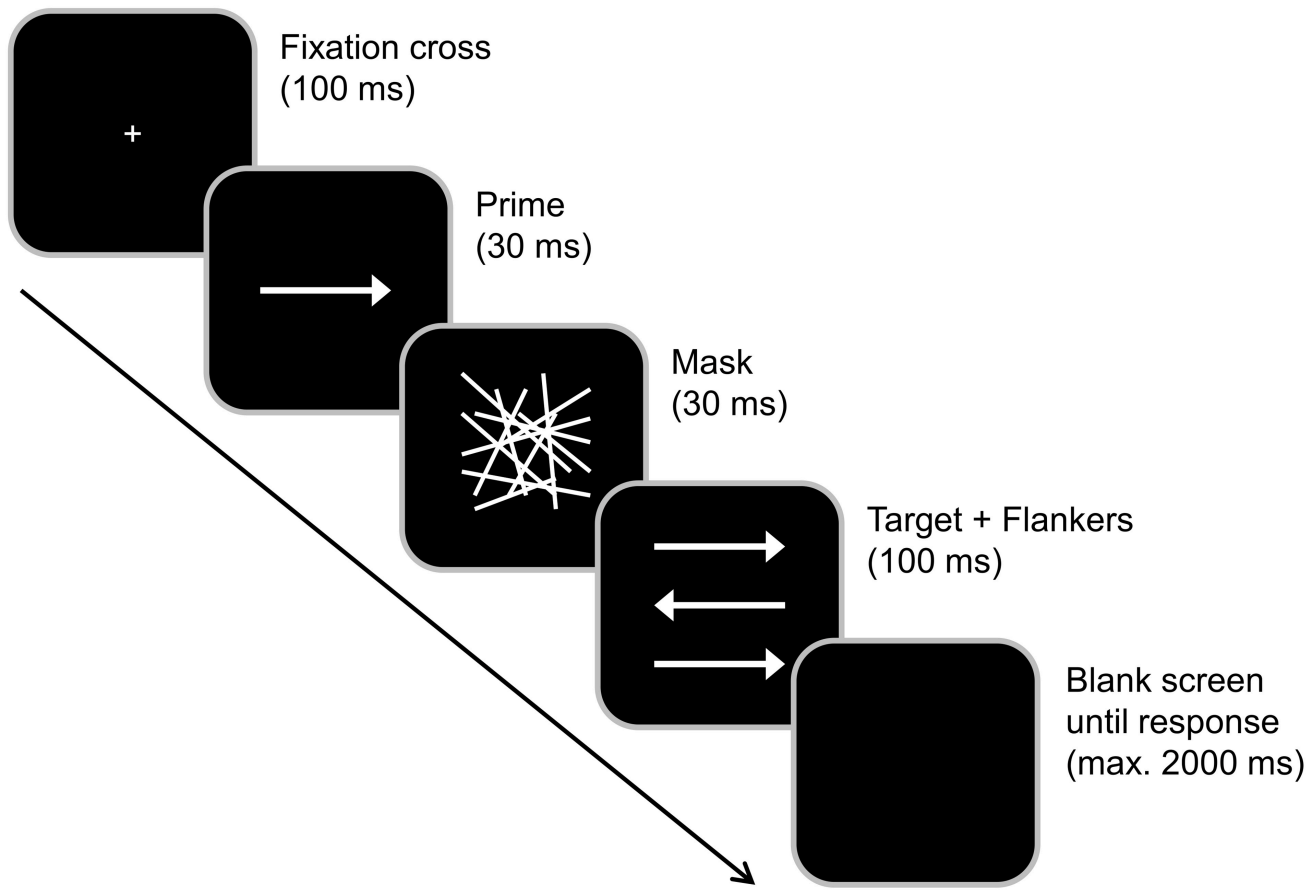
Illustration of the combined conflict paradigm adapted from Boy et al. (2010), as used in our previous acute intoxication study (Stock et al., 2017). Each trial began with a 100 ms presentation of a white fixation cross in the center of a black screen. This was followed by a 30 ms presentation of the prime (a central white arrow) and a 30 ms central presentation of a mask. The target and flankers were simultaneously presented for 100 ms in the center of the screen. Each trial ended either with the first given response indicating the pointing direction of the target stimulus (the arrow in the middle) or 2,000 ms after the onset of the target (in case no responses were given). Compatible and incompatible primes as well as congruent and incongruent flankers were presented equally often and in a random order. The response-stimulus or inter-trial interval randomly varied between 1,000 and 1,200 ms.

Each trial started with a central presentation of a white fixation cross on a black background for 100 ms (see Figure 3 for illustration). Next, a prime (a central white arrow pointing either left or right) was presented for 30 ms. The prime was immediately followed by a central mask (an array of randomly distributed white lines) which was presented for 30 ms, thus producing a stimulus onset asynchrony of 60 ms between prime an target onset. Subsequently, the target (a central white arrow pointing either left or right) and two flankers (white arrows located above and below the target) were simultaneously presented for 100 ms. Participants were asked to indicate the pointing direction of the central target arrow by pressing the left Crtl key with their left index finger in case the target pointed left and by pressing the right Ctrl key with the right index finger in case the target pointed right. Each trial ended either with the first given response or 2,000 ms after the onset of the target (in case no responses were given). In the latter case, the response was coded as a “miss.” The response-stimulus-interval between the participants' first response and the onset of the following trial randomly varied between 1,000 and 1,200 ms.

If the prime and target pointed in the same direction, the trial was classified as compatible. In case when prime and target pointed in opposite directions, the trial was classified as incompatible. When the flankers and target pointed in opposite directions, the trial was classified as incongruent. In case the flankers and target pointed in the same direction, the trial was classified as congruent. Each participant completed 384 trials that were subdivided into 4 blocks. All possible combinations of prime compatibility, flanker congruency and target pointing direction occurred with equal frequency and their order was randomized within each block. In total, the experiment took ~15 min to complete.

After completing the task, the participants were asked whether they had consciously perceived the prime stimulus (i.e., whether they had consciously perceived any visual stimulus preceding the mask, which we termed “scrambled lines” for the sake of better understanding). This was denied by all of them and matches the reports by Boy et al. (2010) who reported no conscious perception of the prime at a SOA of 70 ms (i.e., even 10 ms longer than in our study).

### Statistics

In order to exclude trials with premature responses and to reduce the effect of outliers on mean hit RTs, only correct trials with response times (RTs) between 100 and 1,000 ms were included in the analyses for both tasks. Accuracy and RTs on hit trials were separately analyzed. Hangover state (hangover vs. sober/non-hangover) and flanker congruency (congruent vs. incongruent) were used as within-subject factors in the analyses of both tasks. Flanker congruency of the previous trial (*n*−1 congruent vs. *n*−1 incongruent) was used as an additional within-subject factor in add-on analyses of task 1 (Eriksen Flanker task), while prime compatibility (compatible vs. incompatible) was used as another within-subject factor in task 2 (combined conflict paradigm). The degrees of freedom were adjusted using Greenhouse-Geisser correction and results were Bonferroni-corrected, whenever necessary. For all descriptive statistics, the mean value and standard error of the mean (SEM) is given as a measure of variability.

## Results

### Sample characteristics

After data collection, we had to exclude *n* = 3 participants from the sample as they had shown considerable signs of alcohol tolerance during the intoxication session. They further continued to drink after being released from the laboratory, so that they presented with residual BAC values between 0.7 and 1.1‰ when being assessed the morning after the intoxication session. Hence, *n* = 22 participants remained in the sample. Sociodemographic characteristics, intoxication-related data and subjective hangover ratings of the entire group and of the participants included in the analyses of the two tasks are detailed in Tables 1, 2.

**Table 1:** Sociodemographic and intoxication-related data.

	**Entire sample (*n* = 22)**	**Eriksen flanker task sample (*n* = 16)**	**Combined conflict task sample (*n* = 20)**
Age in years	21.5 ± 0.5	21.6 ± 0.7	21.8 ± 0.5
	range 18–27	range 18 – 27	range 18–27
Cigarettes smoked per day	1.1 ± 0.5	0.8 ± 0.4	1.2 ± 0.6
	range 0–10	range 0–6	range 0–10
Hours of sport per week	4.1 ± 0.6	3.7 ± 0.7	3.9 ± 0.6
	range 0–10	range 0–8	range 0–10
BDI score	3.4 ± 0.7	3.5 ± 0.9	3.6 ± 0.8
	range 0–12	range 0–12	range 0–12
ASI score	11.8 ± 1.3	11.2 ± 1.7	12.2 ± 1.4
	range 2–22	range 2–22	range 2–22
Height in cm	183.6 ± 1.5	185.6 ± 1.5	183.9 ± 1.7
	range 167–198	range 175–198	range 167–198
Weight in kg	78.6 ± 2.4	81.5 ± 2.6	78.9 ± 2.6
	range 56.5–96.5	range 58.5–96.5	range 56.5–96.5
Individual alcohol amount in ml brandy (36 Vol %)	427 ± 8	437 ± 9	427 ± 9
	range 349–497	range 355–497	range 349–497
Consumption duration in min.	179 ± 6	178 ± 8	181 ± 6
	range 115–230	range 115–230	range 115–230
BAC 30 min after end of consumption	1.13 ± 0.04	1.12 ± 0.06	1.13 ± 0.05
	range 0.75–1.5	range 0.75–1.5	range 0.75–1.5
BAC 60 min after end of consumption	1.07 ± 0.05	1.05 ± 0.06	1.07 ± 0.05
	range 0.65–1.5	range 0.65–1.5	range 0.65–1.5
BAC 90 min after end of consumption	1.02 ± 0.03	1.01 ± 0.04	1.01 ± 0.04
	range 0.65–1.26	range 0.65–1.26	range 0.65–1.26
BAC 120 min after end of consumption	0.94 ± 0.03	0.91 ± 0.04	0.92 ± 0.03
	range 0.67–1.16	range 0.67–1.16	range 0.67–1.14

*BDI, beck depression inventory; ASI, axiety sensitivity index; BAC, breath alcohol concentration*.

**Table 2:** Sleep and hangover symptoms on both appointments.

	**Entire sample (*****n*** = **22)**			**Eriksen flanker task sample (*****n*** = **16)**			**Combined conflict task sample (*****n*** = **20)**		
	**Sober**	**Hangover**	**Difference**	**Sober**	**Hangover**	**Difference**	**Sober**	**Hangover**	**Difference**
Hours of sleep in previous night	8.16 ± 0.3	6.03 ± 0.19	*p* < 0.001^**^	8.31 ± 0.34	6.17 ± 0.25	*p* < 0.001^**^	7.98 ± 0.3	6.11 ± 0.2	*p* < 0.001^**^
	range 5.5–10	range 4.5–8		range 5.5–10	range 4.5–8		range 5.5–10	range 4.5–8	
Overall hangover severity	0 ± 0	3.6 ± 0.4	*p* < 0.001^**^	0.1 ± 0.1	3.2 ± 0.5	*p* < 0.001^**^	0.1 ± 0.1	3.8 ± 0.4	*p* < 0.001^**^
	range 0–1	range 0–8		range 0–1	range 0–8		range 0–1	range 1–8	
Headache	0.1 ± 0.1	2.6 ± 0.6	*p* < 0.001^**^	0.1 ± 0.1	2.1 ± 0.7	*p* < 0.009^**^	0.1 ± 0.1	2.7 ± 0.6 r	*p* = 0.001 ^**^
	range 0–1	range 0–9		range 0–1	range 0–6		range 0–1	range 0–9	
Nausea	0 ± 0	1.5 ± 0.4	*p* = 0.001^**^	0 ± 0	1.3 ± 0.4	*p* = 0.010^***^	0 ± 0	1.5 ± 0.4	*p* = 0.001
	range 0–0	range 0–6		range 0–0	range 0–6		range 0–0	range 0–6	
Concentration problems	0.3 ± 0.1	3.6 ± 0.5	*p* < 0.001^**^	0.4 ± 0.2	3 ± 0.6	*p* = 0.001^**^	0.4 ± 0.2	3.9 ± 0.6	*p* < 0.001^**^
	range 0–2	range 0–8		range 0–2	range 0–7		range 0–2	range 0–8	
Regret	0 ± 0	0.4 ± 0.3	*p* = 0.154	0 ± 0	0.6 ± 0.4	*p* = 0.155	0 ± 0	0.5 ± 0.3	*p* = 0.154
	range 0–0	range 0–6	ns.	range 0–0	range 0–6	ns.	range 0–0	range 0–6	ns.
Sleepiness	0.3 ± 0.1	3.7 ± 0.5	*p* < 0.001^**^	0.4 ± 0.2	3.4 ± 0.7	*p* = 0.001^**^	0.4 ± 0.2	3.8 ± 0.6	*p* < 0.001^**^
	range 0–2	range 0–9		range 0–2	range 0–9		range 0–2	range 0–9	
Heart pounding	0.1 ± 0.1	1.5 ± 0.4	*p* = 0.002^**^	0.1 ± 0.1	0.9 ± 0.4	*p* = 0.075	0.1 ± 0.1	1.5 ± 0.4	*p* = 0.004^**^
	range 0–2	range 0–6		range 0–2	range 0–6	ns.	range 0–2	range 0–6	
Vomiting	0 ± 0	0.9 ± 0.3	*p* = 0.008^**^	0 ± 0	0.9 ± 0.4	*p* = 0.029^*^	0 ± 0	0.9 ± 0.3	*p* = 0.015^*^
	range 0–0	range 0–5		range 0–0	range 0–5		range 0–0	range 0–5	
Tired	0.4 ± 0.2	4.3 ± 0.5	*p* < 0.001^**^	0.4 ± 0.2	3.8 ± 0.7	*p* = 0.001^**^	0.5 ± 0.2	4.3 ± 0.6	*p* < 0.001^**^
	range 0–3	range 0–9		range 0–3	range 0–9		range 0–3	range 0–9	
Shivering	0 ± 0	1.1 ± 0.3	*p* < 0.001^**^	0 ± 0	0.7 ± 0.2	*p* = 0.007^**^	0 ± 0	1.1 ± 0.2	*p* < 0.001^**^
	range 0–0	range 0–4		range 0–0	range 0–2		range 0–0	range 0–3	
Clumsy	0.1 ± 0.1	2 ± 0.4	*p* < 0.001^**^	0.1 ± 0.1	1.4 ± 0.4	*p* = 0.002^**^	0.1 ± 0.1	2.1 ± 0.4	*p* < 0.001^**^
	range 0–2	range 0–6		range 0–2	range 0–4		range 0–2	range 0–6	
Weakness	0 ± 0	2.5 ± 0.4	*p* < 0.001^**^	0 ± 0	2.2 ± 0.6	*p* = 0.002^**^	0.1 ± 0.1	2.6 ± 0.5	*p* < 0.001^**^
	range 0–1	range 0–7		range 0–0	range 0–7		range 0–1	range 0–7	
Dizziness	0 ± 0	2.4 ± 0.5	*p* < 0.001^**^	0 ± 0	2.2 ± 0.6	*p* = 0.004^**^	0 ± 0	2.5 ± 0.6	*p* < 0.001^**^
	range 0–0	range 0–8		range 0–0	range 0–8		range 0–0	range 0–8	
Apathy	0.1 ± 0.1	1.3 ± 0.4	*p* = 0.007^**^	0.1 ± 0.1	1.1 ± 0.4	*p* = 0.052	0.1 ± 0.1	1.3 ± 0.4	*p* = 0.012^*^
	range 0–2	range 0–6		range 0–2	range 0–6	ns.	range 0–2	range 0–6	
Sweating	0.1 ± 0.1	1 ± 0.3	*p* = 0.009^**^	0.1 ± 0.1	0.7 ± 0.4	*p* = 0.116	0.1 ± 0.1	1.1 ± 0.4	*p* = 0.013^*^
	range 0–1	range 0–6		range 0–1	range 0–6	ns.	range 0–1	range 0 - 6	
Stomach pain	0 ± 0	0.5 ± 0.3	*p* = 0.094	0.1 ± 0.1	0.3 ± 0.3	*p* = 0.485	0.1 ± 0.1	0.5 ± 0.3	*p* = 0.176
	range 0–1	range 0–4	n.s.	range 0–1	range 0–4	n.s.	range 0–1	range 0–4	n.s.
Confusion	0 ± 0	1.2 ± 0.3	*p* < 0.001^**^	0 ± 0	0.8 ± 0.3	*p* < 0.010^*^	0 ± 0	1.2 ± 0.3	*p* < 0.001^**^
	range 0–0	range 0–4		range 0–0	range 0–4		range 0–0	range 0–4	
Sensitivity to light	0 ± 0	1.7 ± 0.5	*p* = 0.002^**^	0.1 ± 0.1	1.2 ± 0.5	*p* = 0.029^*^	0.1 ± 0.1	1.9 ± 0.5	*p* = 0.002^**^
	range 0–1	range 0–6		range 0–1	range 0–6		range 0–1	range 0–6	
Thirst	0.2 ± 0.1	3.9 ± 0.5	*p* < 0.001^**^	0.3 ± 0.1	3.8 ± 0.6	*p* < 0.001^**^	0.3 ± 0.1	3.7 ± 0.5	*p* < 0.001^**^
	range 0–2	range 1–10		range 0–2	range 1–10		range 0–2	range 1–10	
Heart racing	0 ± 0	0.5 ± 0.2	*p* = 0.009^**^	0 ± 0	0.3 ± 0.1	*p* = 0.104	0 ± 0	0.5 ± 0.2	*p* = 0.008^**^
	range 0–0	range 0–2		range 0–0	range 0–2	ns.	range 0–0	range 0–2	
Anxiety	0.1 ± 0.1	0.5 ± 0.2	*p* = 0.038^*^	0.1 ± 0.1	0.4 ± 0.2	*p* = 0.173	0.1 ± 0.1	0.6 ± 0.2	*p* = 0.038^*^
	range 0–1	range 0–3		range 0–1	range 0–3	ns.	range 0–1	range 0–3	
Depression	0 ± 0	0.7 ± 0.3	*p* = 0.042^*^	0 ± 0	0.8 ± 0.4	*p* = 0.104	0 ± 0	0.8 ± 0.4	*p* = 0.042^*^
	range 0–0	range 0–7		range 0–0	range 0–7	ns.	range 0–0	range 0–7	
Reduced appetite	0 ± 0	1.5 ± 0.4	*p* = 0.004^**^	0.1 ± 0.1	1.7 ± 0.6	*p* = 0.013^*^	0.1 ± 0.1	1.7 ± 0.5	*p* = 0.003^**^
	range 0–1	range 0–7		range 0–1	range 0–7		range 0–1	range 0 – 7	

*All hangover items were taken from van Schrojenstein Lantman et al. (2017c) and rated on a Likert scale ranging from 0 (no symptoms) to 10 (extreme symptoms). Please note that we encouraged participants to accurately rate the severity of each symptom on both appointments even though they had not been drinking the night before the sober appointment. The fact that most participants claimed to have no hangover symptoms in any of the categories on the day of sober testing (and therefore produced minimal variance in the sober ratings) might however have contributed to the observation that the uncorrected paired t-tests reported in the “difference” columns found almost all hangover symptoms to significantly differ between the sober and the hangover appointment*.

* *p < 0.05*,

** *p < 0.01*.

#### Behavioral performance in the eriksen flanker task: general effects

**Figure 4:**
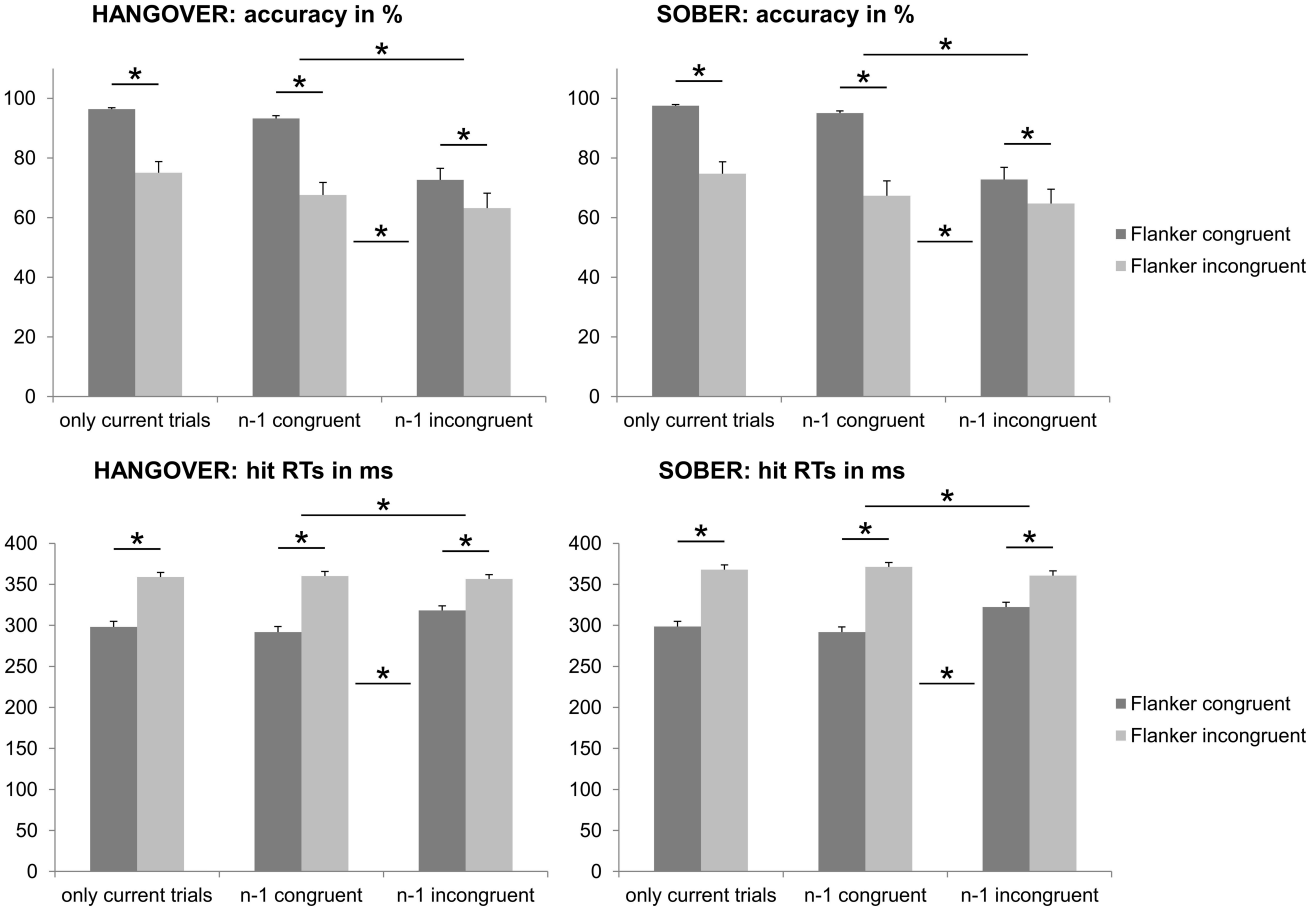
Illustration of the behavioral results obtained with the Eriksen Flanker task. We observed a flanker effect in both behavioral measures, which was reflected by significantly decreased accuracy and increased RTs in case of incompatible flankers, as compared to compatible flankers (see left column of all graphs). We furthermore found the Gratton effect (i.e., larger flanker effects when the *n*−1 trial was congruent, as compared to incongruent) in both behavioral measures (see middle and right column of the graphs). The hangover status did however not modulate any of those measures. Please note that in the upper graphs, the percentages in the left column of each graph are higher as all trials with a correct response were included, whereas both the current and previous (i.e., *n*−1) trial had to have a correct response to be included in the analyses depicted in the middle and right columns of the upper graphs. Error bars denote the standard error of the mean (SEM). Asterisks denote significant effects.

From the *n* = 22 subjects that remained in the sample, *n* = 6 subjects had to be excluded from the analyses of the flanker data due to showing accuracy below chance levels (i.e., <50%) in at least one of the experimental conditions during either their first appointment and/or their sober appointment. The behavioral data of the Eriksen Flanker task are illustrated in Figure 4.

Repeated-measures ANOVAs for the analysis of the accuracy data (hits in percent) showed a main effect of flanker congruency [*F*_(15, 1)_ = 44.307; *p* < 0.001; $\eta_{p}^{2}$ = 0.747], with higher accuracy in the congruent condition (97.1% ± 0.4) than in the incongruent condition (77.0% ± 3.1). There was however no significant main or interaction effect of the hangover status (no hangover vs. hangover) (all *F* ≤ 2.423; *p* ≥ 0.140).

Repeated-measures ANOVAs for the analysis of the hit RT data (in ms) showed a main effect of flanker congruency [*F*_(15, 1)_ = 125.141; *p* < 0.001; $\eta_{p}^{2}$ = 0.893], with faster responses in the congruent condition (301.3 ms ± 5.5) than in the incongruent condition (363.9 ms ± 5.6). There was however no significant main or interaction effect of the hangover status (no hangover vs. hangover) (all *F* ≤ 1.290; *p* ≥ 0.274).

#### Behavioral performance in the eriksen flanker task: gratton effect

In order to assess potential Gratton effects, we ran add-on analyses assessing the influence of the condition in the *n*−1 trial when both the current and the previous trials had correct responses. The Gratton effect is also illustrated in Figure 4.

Repeated-measures ANOVAs for the analysis of the accuracy data (hits in percent) again showed a main effect of flanker congruency in the current trial [*F*_(15, 1)_ = 53.959; *p* < 0.001; $\eta_{p}^{2}$ = 0.782; congruent = 84.7% ± 1.8; incongruent = 67.6% ± 4.0], as well as a main effect of flanker congruency in the previous (*n*−1) trial [*F*_(15, 1)_ = 47.805; *p* < 0.001; $\eta_{p}^{2}$ = 0.761; congruent = 81.7% ± 2.1; incongruent = 70.7% ± 3.6]. There was furthermore a significant interaction of the flanker congruency in the previous and current trials [*F*_(15, 1)_ = 32.259; *p* < 0.001; $\eta_{p}^{2}$ = 0.683]. *Post-hoc* paired *t*-tests showed that while all four possible contrasts were significant (all *t* ≥ 3.042; *p* ≤ 0.008; CC = 94.3% ± 0.7; IC = 75.1% ± 3.2; CI = 69.0% ± 3.9; II = 66.2% ± 4.1), there was a significant Gratton effect: The flanker effect (congruent minus incongruent) was significantly larger when the *n*−1 trial was congruent (25.3% ± 3.7) than when the *n*−1 trial was incongruent (8.9% ± 1.3) [*t*_(15)_ = 5.680; *p* < 0.001]. There was however no significant main or interaction effect of the hangover status (no hangover vs. hangover) (all *F* ≤ 2.070; *p* ≥ 0.171).

Repeated-measures ANOVAs for the analysis of the hit RT data (in ms) again showed a main effect of flanker congruency in the current trial [*F*_(15, 1)_ = 97.806; *p* < 0.001; $\eta_{p}^{2}$ = 0.867; congruent = 308.9 ms ± 4.8; incongruent = 362.9 ± 5.5], as well as a main effect of flanker congruency in the previous (*n*−1) trial [*F*_(17, 1)_ = 54.386; *p* < 0.001; $\eta_{p}^{2}$ = 0.784; congruent = 330.5 ms ± 4.7; incongruent = 341.2 ms ± 4.2]. There was furthermore a significant interaction of the flanker congruency in the previous and current trials [*F*_(17, 1)_ = 63.764; *p* < 0.001; $\eta_{p}^{2}$ = 0.810]. Matching the observations made for the accuracy measures, *post-hoc* paired *t*-tests showed that while all four possible contrasts were significant (all *t* ≥ 2.768; *p* ≤ 0.014; CC = 294.5 ms ± 5.8; IC = 323.2 ms ± 4.1; CI = 366.5 ms ± 5.6; II = 359.2 ms ± 5.7), there was a significant Gratton effect: The flanker effect (incongruent minus congruent) was significantly larger when the *n*−1 trial was congruent (72.2 ms ± 6.3) than when the *n*−1 trial was incongurent (36.0 ms ± 5.4) [*t*_(15)_ = 7.985; *p* < 0.001]. There was however no significant main or interaction effect of the hangover status (no hangover vs. hangover) (all *F* ≤ 4.359; *p* ≥ 0.054).

#### Behavioral performance in the combined conflict paradigm: general effects

**Figure 5:**
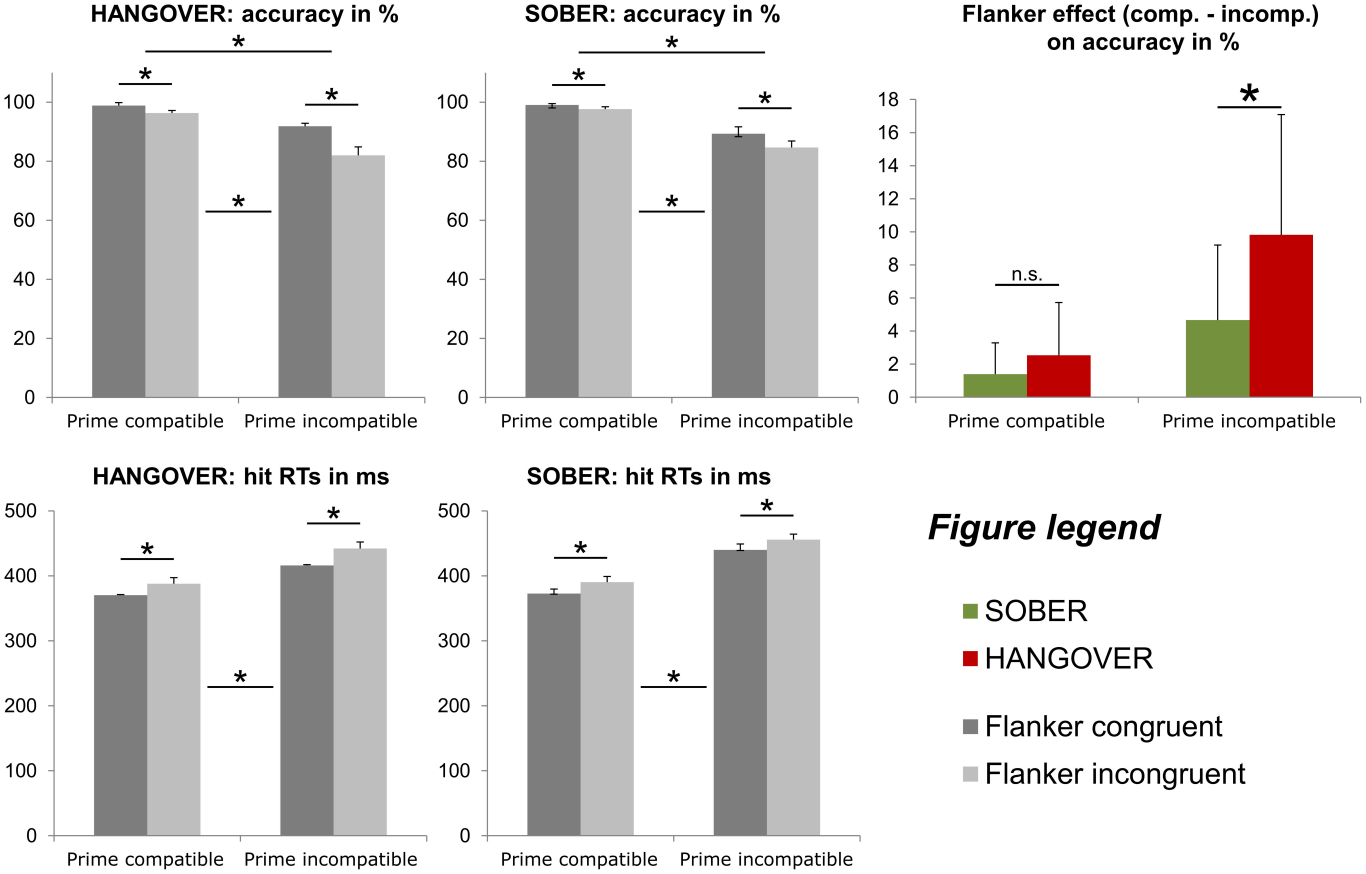
Illustration of the behavioral results obtained with the conflict paradigm adapted from Boy et al. (2010). In line with the findings of previous publications on this paradigm, both types of distractors (subliminal primes and consciously processed flankers) induced a significant conflict, as indicated by decreased accuracy (top row) and increased response times (bottom row). Furthermore, subliminal conflict load (i.e., incompatible primes) increased the flanker effect on accuracy (top row). With respect to hangover effects, we found that hungover participants displayed a significantly larger flanker effect, but only when the subliminal prime was incompatible (see colored graph on the right). Error bars denote the standard error of the mean (SEM). Asterisks denote significant effects (for practical reasons, hangover effects are only denoted in the very right graph).

From the *n* = 22 subjects that remained in the sample, *n* = 2 subjects were excluded from the behavioral analyses data due to showing accuracy close to or below chance level (i.e., <55%) in at least one of the experimental conditions during either their first appointment and/or their sober appointment. Behavioral data of the combined conflict paradigm are illustrated in Figure 5.

Repeated-measures ANOVAs for the analysis of the accuracy data (hits in percent) showed significant main effects of both prime compatibility [*F*_(19, 1)_ = 34.897; *p* < 0.001; $\eta_{p}^{2}$ = 0.647; compatible = 98.0% ± 0.6; incompatible = 86.9% ± 2.1] and flanker congruency [*F*_(19, 1)_ = 47.658; *p* < 0.001; $\eta_{p}^{2}$ = 0.715; congruent = 94.8% ± 1.1; incongruent = 90.1% ± 1.3]. In line with previous studies using this paradigm (e.g. Stock et al., 2016b, 2017; Gohil et al., 2017), we also found a significant interaction of prime compatibility ^*^ flanker congruency [*F*_(19, 1)_ = 23.704; *p* < 0.001; $\eta_{p}^{2}$ = 0.555]. Most importantly, however, we found a significant interaction of hangover state ^*^ flanker congruency [*F*_(19, 1)_ = 12.371; *p* = 0.002; $\eta_{p}^{2}$ = 0.394] as well as an interaction of hangover state ^*^ prime compatibility ^*^ flanker congruency [*F*_(19, 1)_ = 6.832; *p* = 0.017; $\eta_{p}^{2}$ = 0.264]. *Post-hoc* ANOVAs that were separately conducted for both prime conditions showed that in case of compatible primes, there was only a main effect of flanker congruency [*F*_(19, 1)_ = 17.151; *p* = 0.001; $\eta_{p}^{2}$ = 0.474; congruent = 98.9% ± 0.4; incongruent = 97.0% ± 0.8], but no main or interaction effect of hangover state (all *F* ≤ 3.130; *p* ≥ 0.093). In contrast to this, trials with incompatible primes showed an main effect of flanker congruency [*F*_(19, 1)_ = 41.911; *p* < 0.001; $\eta_{p}^{2}$ = 0.688; congruent = 90.6% ± 2.0; incongruent = 83.3% ± 2.3] as well as an interaction of hangover state ^*^ flanker congruency [*F*_(19, 1)_ = 11.500; *p* = 0.003; $\eta_{p}^{2}$ = 0.377]. Further *post-hoc* paired *t*-tests in trials with incompatible primes showed that while there was a significant flanker congruency effect both during the sober and hangover appointment (all *t* ≥ 4.614; *p* < 0.001), the flanker effect (congruent minus incongruent) was significantly larger during the hangover appointment (9.8% ± 1.6) than during the sober appointment (4.7% ± 1.0). Given that not all accuracy variables were normally distributed, we further confirmed this finding with a Wilcoxon-signed rank test for paired samples, which further underpinned this finding (*p* = 0.002). We further conducted add-on analyses to investigate whether there were any effects of appointment order on the size or direction of the observed hangover effect. Wilcoxon-signed rank tests for paired samples showed that both sub-groups had significantly larger flanker effects during the hangover than the sober appointment in case of incompatible primes (sober first group: *p* = 0.033; hangover first group: *p* = 0.21). The lack of order effects was further underlined by the finding that the size of this effect did not significantly differ between the order sub-groups, as evidenced by a Mann–Whitney-U-test for independent samples (*p* = 0.766). All other main or interaction effects of the ANOVA on accuracy data were non-significant (all *F* ≤ 0.161; *p* ≥ 0.693).

Repeated-measures ANOVAs for the analysis of the hit RT data (in ms) showed significant main effects of both prime compatibility [*F*_(19, 1)_ = 181.291; *p* < 0.001; $\eta_{p}^{2}$ = 0.905; compatible = 380.2 ms ± 6.8; incompatible = 438.4 ± 7.0] and flanker congruency [*F*_(19, 1)_ = 66.220; *p* < 0.001; $\eta_{p}^{2}$ = 0.777; congruent = 399.7 ms ± 6.4; incongruent = 418.9 ms ± 6.9]. All other main or interaction effects of the ANOVA on hit RTs failed to reach significance (all *F* ≤ 3.921; *p* ≥ 0.062).

Of note, we refrained from assessing the Gratton effect in this paradigm as we had already assessed it in the “regular” flanker task and further adding this factor in the analyses would have substantially increased the number of condition combinations to a level where we might have encountered power issues due to our sample size.

#### Behavioral performance in the combined conflict paradigm: linear correlations

**Figure 6:**
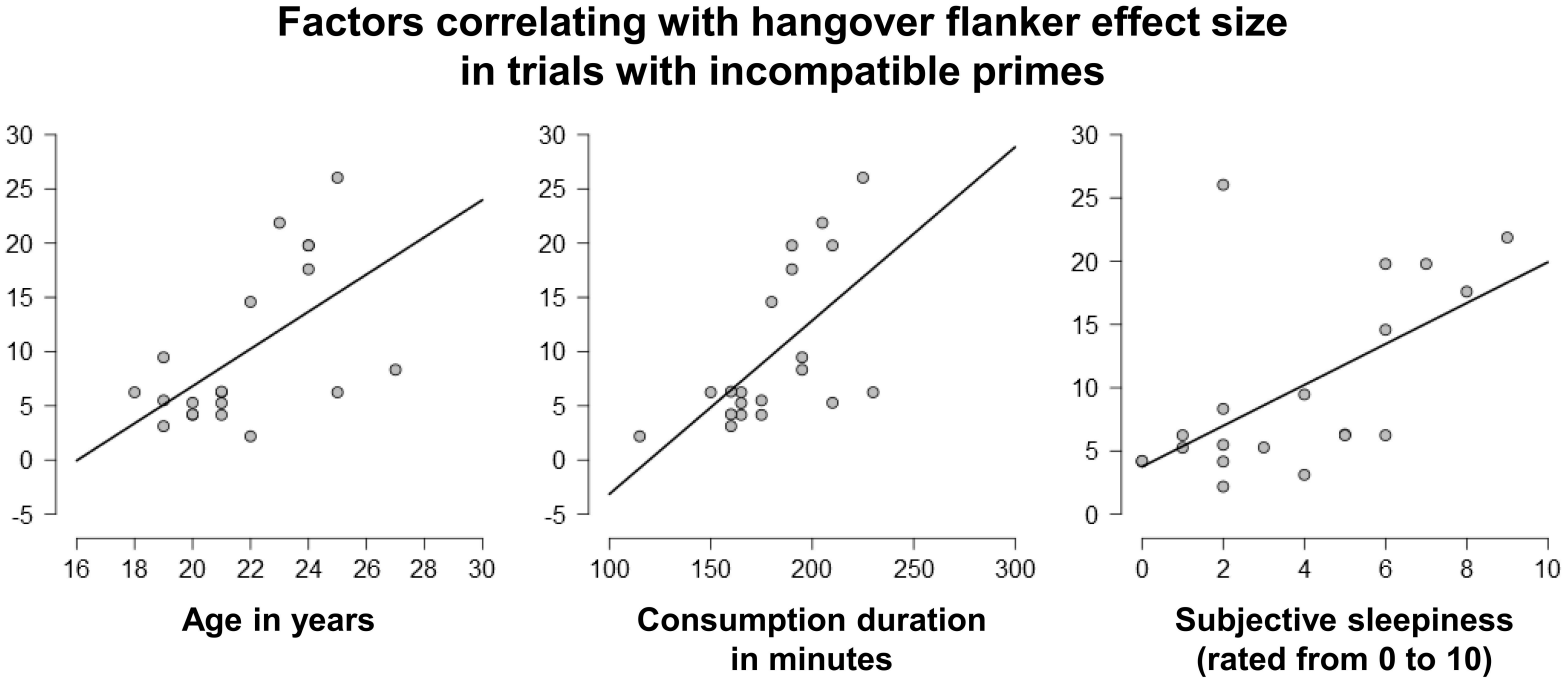
Linear correlation analyses. The size of the flanker effect, which had been found to be increased by hangover when the subliminal primes were incompatible, was positively correlated with age **(left)**, consumption speed during intoxication induction **(middle)**, and subjective sleepiness during the hangover appointment **(right)** (all *p* < 0.010). It should however be noted that age and consumption speed were also positively correlated.

After finding that alcohol hangover significantly increased the flanker effect when the target was preceded by an incompatible prime (i.e., when conflict load was high), we conducted linear correlation analyses to see whether the size of the flanker effect in the hangover and/or sober condition was associated with any of the assessed sociodemographic or alcohol-related factors (see Tables 1, 2). Correlations with a *p* < 0.010 are illustrated in Figure 6.

When not correcting for multiple comparisons, we found positive correlations between the hangover flanker effect size in prime-incompatible trials and age in years [*r*_(20)_ = 0.579; *p* = 0.008] as well as consumption duration in minutes [*r*_(20)_ = 0.612; *p* = 0.004]. It should however be noted that those two factors were inter-correlated [*r*_(20)_ = 0.539; *p* = 0.014], as older participants tended to drink more slowly than younger ones. The effects observed for drinking speed might therefore be due to age as a potential confound. We furthermore found positive correlations between the hangover flanker effect size in prime-incompatible trials and the BDI score [*r*_(20)_ = 0.461; *p* = 0.041] and the ASI score [*r*_(20)_ = 0.467; *p* = 0.038]. Importantly, none of those four factors significantly correlated with the sober flanker effect size in prime-incompatible trials (all *p* ≥ 0.154). We also found no correlation of height, weight, the absolute amount of consumed alcohol, BAC levels during intoxication, cigarette consumption, or sport habits with either the hangover, or the sober flanker effect in trials with incompatible primes (all *p* ≥ 0.118).

We furthermore ran separate correlation analyses for both appointments in order to relate appointment-specific ratings of sleep duration and hangover symptoms as listed in van Schrojenstein Lantman et al. (2017c) to the flanker effect obtained during the respective appointments (again, the reported values are not corrected for multiple comparisons). For the hangover appointment, we found positive correlations between the hangover flanker effect size in prime-incompatible trials and the subjective rating of sleepiness [*r*_(20)_ = 0.591; *p* = 0.006], but not with tiredness [*r*_(20)_ = 0.325; *p* = 0.162] or the hours slept in the night prior to the hangover appointment [*r*_(20)_ = 0.372; *p* = 0.107]. Interestingly, there was also no correlation between sleep duration and subjective sleepiness [*r*_(20)_ = −0.138; *p* = 0.561]. We furthermore found positive correlations of the hangover flanker effect size in prime-incompatible trials with the subjective rating of sweating [*r*_(20)_ = 0.538; *p* = 0.014], feelings of depression [*r*_(20)_ = 0.466; *p* = 0.038], and reduced appetite [*r*_(20)_ = 0.514; *p* = 0.020]. None of the other hangover-associated symptoms, including the subjective overall rating of hangover severity, showed significant correlations (all *p* ≥ 0.102). During the sober appointment, there was again no significant correlation between the behavioral measure and sleep duration or any of the assessed subjective hangover symptoms (all *p* ≥ 0.183), but it should also be noted that all hangover symptoms received very low average ratings during the sober appointment (see Table 2).

In order to further investigate whether the observed hangover effects were strongly influenced by age, consumption duration, and sleepiness (i.e., all correlations with a *p* < 0.010), we ran two separate analyses: The first was a univariate ANCOVA investigating whether any of the three covariates could explain a significant amount of the inter-individual variance of the hangover flanker effect size in prime-incompatible trials. This was not the case (all *F* ≤ 4.182; *p* ≥ 0.058). The second analysis was a repeated measures ANCOVA for Flanker effect size in trials with incompatible primes using hangover state as a within subject factor and all three correlates as covariates. The inner-subject effects of this analysis showed that all three factors and their interaction significantly modulated the hangover effect (i.e., difference between sobriety and hangover) in Flanker effect size of trials with incompatible primes (all *F* ≥ 5.068; *p* ≤ 0.044). But while the hangover effect became somewhat smaller, it still remained significant when correcting for all of those covariates [*F*_(12,1)_ = 5.227; *p* = 0.040; $\eta_{p}^{2}$ = 0.305; corrected hangover flanker effect in incompatible primes = 9.4% ± 1.2; corrected sober flanker effect in incompatible primes = 5.1% ± 1.5].

### Result overview: hangover effects

We found that the size of consciously perceived stimulus conflicts (i.e., the flanker effect) was not significantly increased during hangover unless the target was preceded by an incompatible subliminal prime. The size of this effect appeared to be strongly correlated (i.e., *r* ≥ 0.5) with the inter-correlated factors of age and alcohol consumption speed, and the subjective rating of sleepiness, sweating and reduced appetite during the hangover appointment. Yet still, the observed hangover effect remained significant when controlling for the most important three factors as covariates. Moderate correlations (i.e., 5 > *r* ≥ 0.3) were found for depressive symptoms, anxiety sensitivity and the subjective rating of feelings of depression during the hangover appointment. Yet, those correlations should be treated with ample caution as we ran a very large number of uncorrected multiple comparisons.

## Discussion

Given our previous finding that alcohol hangover may increase the drift rate (i.e., the speed of task/response-relevant information accumulation from visual stimuli; Stock et al., 2016b), we investigated whether it increases the processing of masked subliminal distractors and thereby modulates top-down control processes via conflict load. In line with the recommendation made by Stephens et al. (2014), we assessed top-down executive functioning with a flanker task. We further used a combined conflict paradigm introduced by Boy et al. (2010) to precede a consciously perceived combination of target and flanker stimuli with subliminal masked primes (Boy et al., 2010; Stock et al., 2016a, 2017; Gohil et al., 2017). Each subject was tested twice, i.e., once hangover and once sober (always at a BAC of 0.00‰). Hangover symptoms were experimentally induced by the administration of a standardized amount of brandy and/or red wine the night before the hangover assessment and quantified with the help of subjective ratings on a 0–10 Likert scale.

### Hangover increases subliminal conflict load due to attentional changes

With respect to our main research question, we found that alcohol hangover altered the processing of subliminal information, as reflected by an increased Flanker effect in case of incompatible primes only. This finding is well in line with previous studies using the same experimental paradigm (Stock et al., 2016a, 2017; Gohil et al., 2017), which have also shown a clear interaction of subliminally and consciously processed conflicts (i.e., larger behavioral conflicts/flanker effects whenever the conflict load is increased/incompatible primes are present). Against this background, our results suggest that the conflict load induced by incompatible subliminal primes was significantly larger in the hangover condition and that this hampered top-down control of the consciously perceived flanker-type distractors. When speculating about the mechanisms underlying this effect, it is helpful to take a closer look at how subliminal primes influence behavioral performance: Priming is generally defined as “the influence of one event on performance during a second event” (Klapp, 2015). We used primes that directly bias responding (so-called direct primes). While explicit priming requires conscious awareness of the prime, the associative response priming used in our study also takes place when the prime itself is not phenomenally visible (Klapp, 2015). It may therefore occur without consciousness and it also does not seem to be driven by intentional control over automaticity. Instead, it seems to be heavily based on (self-)automatization of S-R associations (Klapp, 2015). On the neuroanatomical level, the distinction between explicit and associative is reflected by the anterior cingulate cortex (ACC), which has been shown to be activated by explicit primes, but not by subliminal ones (Dehaene et al., 2003; Mayr, 2004). The ACC is known to play a key role for top-down cognitive control, conflict monitoring and task effort (Botvinick et al., 2004; Larson et al., 2014) and has repeatedly been shown to reflect variations in the size of the Flanker effect (Larson et al., 2014; Stock et al., 2017; e.g., Bluschke et al., 2017). As subliminal/associative primes do not seem to modulate activity in this brain region (Dehaene et al., 2003; Mayr, 2004), it does not seem very likely that the observed increase in flanker effect size in trials with incompatible primes was caused by straining conflict monitoring resources. It has however been noted that “despite its invisibility, a masked stimulus if used as a prime can influence a variety of executive functions, such as response activation, semantic processing, or attention shifting” (Ansorge et al., 2014). Semantic information did not need to be processed in our study, but selective attention and response selection both play an important role for behavioral performance in the paradigm we used (Olk et al., 2015): It is generally believed that a failure to fully ignore distractors like flanker stimuli triggers rather automatic response tendencies, because distractors and target share response-relevant stimulus features. Those automatic response tendencies then cause a response conflict in case they do not match the correct, top-down guided response (Olk et al., 2015). There may hence be a conflict at the level of stimulus-driven attention due processing of “task-relevant” stimulus features of the distractors. Additionally, there may be a conflict at the level of response selection due to reduced response selection capacities. In the light of the results we obtained, the latter does however not seem very likely. The reason for this is that if response selection capacities were generally impaired this should also show in general Flanker effect size differences (irrespective of conflict load): Target and flankers are thought to cause response selection conflicts in (pre)motor areas, which are processed and (partly) resolved in fronto-striatal structures, including the ACC (Larson et al., 2014; Olk et al., 2015). Matching this, ACC activity in response to incongruent flankers has also been shown to mediate the Gratton effect (i.e., the attenuation of conflict size following a conflict trial) (Larson et al., 2014). Given that we found no general (i.e., prime-independent) modulation of flanker effect size and no modulation of the Gratton effect by hangover state, we are inclined toward attributing the observed hangover effects to differences in attentional processes, rather than response selection. While this claim certainly needs to be underpinned with further studies, it nicely matches our initial hypothesis that faster processing of visual information during hangover (Stock et al., 2016b) should increase the processing of the briefly presented subliminal primes, thus inducing a greater (subliminal) conflict load.

### Hangover increases prime-induced conflicts, not response facilitation

In the context of the combined conflict paradigm that we employed, it is however important to clearly differentiate between the effect of compatible and incompatible primes. The main reason for this is that in case of the short stimulus onset asynchronies we used, the prime condition differences are usually attributed to the positive compatibility effect (PCE). This effect is commonly assumed to be based on an *improvement* of behavioral performance due to correct automatic response tendencies evokes by compatible primes, rather than a worsening of behavioral performance evoked by incompatible primes (Eimer and Schlaghecken, 2003; Boy et al., 2010). Against this background, it would seem logic to argue that compatible primes facilitate responding, thereby masking hangover-associated increases in the flanker effect rather than expecting an increase in the conflict arising from incompatible primes. Yet, our data refute this idea because we found no hangover modulation of the flanker effect in case of compatible primes or in the “classic” Eriksen Flanker task. Given that the Eriksen Flanker task was designed to maximize the flanker effect and therefore differed from the combined conflict task in several respects (i.e., it employed larger arrowheads, had a larger percentage of compatible trials and induced time pressure), we however refrained from a direct comparison of flanker effect size across both paradigms.

### Conflict monitoring and response selection seem largely unaffected by hangover

Given that two previous studies from another work group have reported alcohol hangover to impair selective attention, as assessed with an Eriksen Flanker task (McKinney et al., 2012a,b), our null finding needs to be discussed. There are however several methodological differences between those two studies and our current study which should be carefully considered: The studies by McKinney et al. (2012a,b) used a naturalistic design where participants were asked to come to the lab after a typical night of drinking and could only provide retrospective reports of their alcohol consumption (which might however have roughly matched our experimental amounts). More importantly, however, those two studies varied the spatial distance between target and flankers and only found a differential modulation of the flanker effect in hit RTs when the distance between flankers and target was large (more than 3 cm), but not when it was small (1 cm, which is comparable to our study). Only in trials with large distances, sober participants showed a smaller flanker effect than hungover participants. The authors interpreted this as a more narrow attentional focus in sober participants whom they interpreted to be better able to exert selective cognitive control. While this interpretation is certainly in line with the common literature, it does not confute our hypothesis on “improved” visual processing—it could well be possible that hungover participants do not only process potentially task-relevant information faster, but they could also be inclined to take more information into account. Stock et al. (2017) have suggested the increment in visual processing to be potentially related to an acetaldehyde-induced decrease in GABAergic signaling. This could provide a starting ground for further studies assessing this question in greater detail—especially given that a direct causal/functional link has not yet been established in human subjects.

### Factors correlating with the hangover-associated increase in (subliminal) conflict load

Trying to assess other factors that might have played a role in the observed behavioral effect, we conducted (uncorrected) linear correlation analyses, where the flanker effect size in trials with incompatible subliminal primes was related to various intoxication and hangover measures. Matching anecdotal evidence on hangovers getting worse as we age, we found that the performance decrease/increase in conflict size during the hangover appointment showed a strong positive correlation with age (even though covariate analyses showed that age did not explain a significant proportion of variance in the hangover measure). This finding was further underpinned by the lack of age effects on the same performance measure as assessed during the sober appointment. While we unfortunately lack any biochemical markers which might explain this effect, it could be speculated that a decrease in the metabolic rate with which ethanol is broken down by the liver mediates this effect: As we age, the metabolization rate of ethanol decreases (Meier and Seitz, 2008) and might therefore increase the concentration of harmful metabolites like methanol and acetaldehyde, which are both known to have strong detrimental effects on cognition, behavior, and well-being. Acetaldehyde is of special interest here as it parallels the dopaminergic effects of ethanol, but has the opposite effect on GABAergic signaling (ethanol increases GABAergic signaling, acetaldehyde decreases it) (Foddai et al., 2004; Melis et al., 2007; Enrico et al., 2009; Correa et al., 2012; Martí-Prats et al., 2013) and has therefore been suspected to underlie the differential modulation of information accumulation speed by acute alcohol intoxication and hangover (Stock et al., 2017). Still, further studies will be needed to investigate this hypothesis. Also, the finding that age (as a covariate) did not explain a significant proportion of variance in the hangover measure and correcting for age as a covariate still left the observed hangover effect on incompatible trials significant.

Aside from age (and the inter-correlated factor of drinking speed), we also found sleepiness to be strongly correlated to our behavioral measure of interest. Among all hangover symptoms assessed by van Schrojenstein Lantman et al. (2017b), sleepiness has been demonstrated to have one of the strongest impacts on cognitive functions and mood. It does however require further studies to clarify whether there is a true functional nexus between sleepiness and cognitive dysfunction or whether those two factors are rather independent and merely modulated by a common underlying neurobiochemical factor like acetaldehyde concentrations (which could potentially also have an influence of the other hangover symptoms that showed a correlation with the performance measure). Of note, it is however unlikely that the subjective feeling of sleepiness reflected the participants' actual lack of sleep as we found no correlation between sleep duration and sleepiness in neither of the two appointments. Furthermore, covariate analyses showed that subjective sleepiness did not explain a significant proportion of variance in the hangover measure or eliminate the hangover effect in incompatible trials when corrected for. Sleepiness may hence be a potentially confounding factor, but does certainly not explain the entirety of the hangover effect we observed.

Lastly, we observed a functional nexus between depression/anxiety scores and hangover-associated performance decrements. While those (uncorrected) correlations should always be treated with ample caution, they might be related to recent findings that depression symptoms seem to be associated with hangover susceptibility (Piasecki et al., 2017). In line with this, the authors of that recent study noted that “hangover and depression overlap symptomatically and are empirically associated with one another, suggesting the possibility that common underlying causal mechanisms may contribute to both phenomena.” Further investigation of this matter might hence prove fruitful.

Another aspect worth discussing is the absence of positive correlations between peak BAC values or the subjective rating of overall hangover severity with the cognitive hangover effects found in our study: Previous studies have provided convincing evidence in favor of the assumption that higher peak (e)BACs make developing a hangover more likely and are furthermore correlated with the subjective severity of hangover symptoms (Kruisselbrink et al., 2017). Yet still, our findings do not necessarily invalidate those studies as we standardized the amount of alcohol, thus trying to minimize variation in hangover severity. Hence, the observed variation in peak BAC values and hangover ratings are likely due to factors other than alcohol consumption itself. Against this background, it may therefore even be expected to not see a strong statistical correlation of those measures with hangover-induced performance impairments. In order to systematically investigate the effects of hangover severity, a larger sample would be needed to assess subgroups that each receive a different amount of alcohol.

### Limitations

There are of course also a few limitations which should be discussed: Due to ethical concerns, we did not have permission to include females in this study. On average, females tend to metabolize alcohol more slowly than males and may furthermore show more pronounced cognitive impairments as a consequence of long-term binge drinking habits (Montgomery et al., 2012). Additionally, it has been suggested that women might process task-irrelevant information/flanker stimuli to a greater extent than men, thus potentially yielding larger Flanker effects (Stoet, 2010; Clayson et al., 2011; Judge and Taylor, 2012; Larson et al., 2014). Against this background, it could be conceivable that cognitive hangover effects on how subliminal conflicts modulate the cognitive control (as assessed via the flanker effect) might on average be more pronounced in females than in males. It would furthermore be possible that only women show general, hangover-related increases in Flanker effect size. Further studies including both sexes will be needed to investigate such claims.

We also did not administer a placebo before the sober assessment, but given that we administered quite large amounts of alcoholic beverages, it would have been quite easy for the participants to tell apart the placebo and alcoholic drinks. Given that the overall performance (both response accuracy and RTs) did not generally differ between the sober and hangover appointments, it does however not seem very likely that the participants unwittingly put less effort during the hangover appointment than during the sober appointment (for elaborating comments on this issue, please see Stephens et al., 2008).

It might furthermore be criticized that we did not counter-balance task order (i.e., ask half of the participants to first perform the Eriksen Flanker task and ask the other half of participants to first perform the combined conflict task). As we however wanted to differentiate between the flanker effect and the effect of subliminal primes onto that phenomenon, we had to exclude the possibility of carryover or expectancy effects and therefore decided to always conduct the Eriksen Flanker task first. Additionally, the two tasks were not conducted in direct succession and differed with respect to the employed stimulus material/condition frequency in order to minimize carry-over effects. Given that add-on analyses of appointment group found no significant differences in the size or direction of the observed hangover effect, it does not seem very likely that performing the Eriksen Flanker task would have yielded an advantage or disadvantage for performing the combined conflict task.

## Conclusions

Given our previous finding that alcohol hangover may increase the speed of information accumulation from visual stimuli, we investigated whether it also increases the processing of masked subliminal distractors and thereby modulates top-down control processes. In line with this, we found that hangover potentiates the detrimental effects of conflicting subliminal primes on top-down cognitive conflicts. The size of this effect was positively correlated with age and subjective sleepiness during the hangover state, but these factors did not mediate the entire effect, as the hangover effect remained significant even after correcting for those covariates. We further found no correlation of the behavioral effect with the subjective overall rating of hangover symptoms or the maximal breath alcohol concentration reached during prior intoxication. This suggests that alcohol hangover may affect cognitive performance due to an increase in non-conscious processing of visual distractors.

## Author contributions

A-KS and NZ collected the data. A-KS analyzed the data. All authors were involved in study design, data interpretation, and writing the final manuscript. All authors approved the final manuscript.

### Conflict of interest statement

The authors declare that the research was conducted in the absence of any commercial or financial relationships that could be construed as a potential conflict of interest.
